# Single amino acids set apparent temperature thresholds for heat-evoked activation of mosquito transient receptor potential channel TRPA1

**DOI:** 10.1016/j.jbc.2022.102271

**Published:** 2022-07-16

**Authors:** Thi Hong Dung Nguyen, Stella Chapman, Makiko Kashio, Claire Saito, Tatjana Strom, Mio Yasui, Makoto Tominaga

**Affiliations:** 1 Division of Cell Signaling, National Institute for Physiological Sciences, National Institutes of Natural Sciences, Okazaki, Japan; 2 Thermal Biology Group, Exploratory Research Center on Life and Living Systems (ExCELLS), National Institutes of Natural Sciences, Okazaki, Japan; 3 Institute for Biology, Humboldt University of Berlin, Berlin, Germany; 4 Nagoya City University Medicine School, Nagoya, Japan

**Keywords:** transient receptor potential channels, insect, physiology, protein chimera, mutagenesis, electrophysiology, *Aa*, *Aedes aegypti*, AR, ankyrin repeat, *As*, *Anopheles stephensi*, *Cp*, *Culex pipiens pallens*, HEK293T, human embryonic kidney–derived 293T cell line, TRP, transient receptor potential, TRPA1, TRP ankyrin 1

## Abstract

Animals detect heat using thermosensitive transient receptor potential (TRP) channels. In insects, these include TRP ankyrin 1 (TRPA1), which in mosquitoes is crucial for noxious heat avoidance and thus is an appealing pest control target. However, the molecular basis for heat-evoked activation has not been fully elucidated, impeding both studies of the molecular evolution of temperature sensitivity and rational design of inhibitors. In TRPA1 and other thermosensitive TRPs, the N-terminal cytoplasmic ankyrin repeat (AR) domain has been suggested to participate in heat-evoked activation, but the lack of a structure containing the full AR domain has hindered our mechanistic understanding of its role. Here, we focused on elucidating the structural basis of apparent temperature threshold determination by taking advantage of two closely related mosquito TRPA1s from *Aedes aegypti* and *Culex pipiens pallens* with 86.9% protein sequence identity but a 10 °C difference in apparent temperature threshold. We identified two positions in the N-terminal cytoplasmic AR domain of these proteins, E417 (*A. aegypti*)/Q414 (*C. pipiens*) and R459 (*A. aegypti*)/Q456 (*C. pipiens*), at which a single exchange of amino acid identity was sufficient to change apparent thresholds by 5 to 7 °C. We further found that the role of these positions is conserved in TRPA1 of a third related species, *Anopheles stephensi*. Our results suggest a structural basis for temperature threshold determination as well as for the evolutionary adaptation of mosquito TRPA1 to the wide range of climates inhabited by mosquitoes.

Animals sense external temperature using thermosensitive transient receptor potential (TRP) channels, polymodal receptors for heat and chemical stimuli, which make up a subclass of the larger TRP channel family (1, 2). In some animals, including insects, TRP ankyrin 1 (TRPA1) is a receptor for noxious high temperature and chemicals (3, 4, 5), but the structural basis of thermosensation has not been fully elucidated.

Critical regions for thermosensation have been suggested (6, 7, 8) within the N-terminal cytoplasmic ankyrin repeat (AR) domain, which is found in each subunit of the TRPA1 homotetramer (9). However, no structure including the full ARs has yet been reported, and the mechanism by which they affect heat-evoked activation remains unknown.

Among TRPA1 homologs, mosquito TRPA1s are an appealing target for study because mosquitoes, whose blood-feeding drive makes them the principal infectious disease vector across tropical and subtropical regions (10), seek hosts by their warm temperatures (11) while depending on TRPA1 thermosensing to identify and avoid noxious high temperatures (4). We recently reported that TRPA1 from the tropical *Aedes aegypti* (*Aa* TRPA1, 32 ± 0.8 °C) has an apparent threshold 10 °C higher than that of the temperate *Culex pipiens pallens* (*Cp* TRPA1, 21.8 ± 0.7 °C), mirroring the behavioral difference in noxious temperature avoidance thresholds in these mosquitoes (12). The unusually high 86.9% protein sequence identity between these homologs provides an opportunity to elucidate structural elements associated with threshold determination, and thereby with environmental adaptation, *via* exchange of domains and single amino acids.

For mosquito TRPA1s, we hypothesized that specific residues in the ARs set the apparent temperature thresholds. Using chimeric channels to narrow down a region of interest, we then identified residues within this region that, when exchanged with their counterpart from the other species, alter channel threshold by 5 to 7 °C. Our results show that single-point mutations are sufficient to allow adaptation of TRPA1 to warmer or colder environments.

## Results

**Figure 1:**
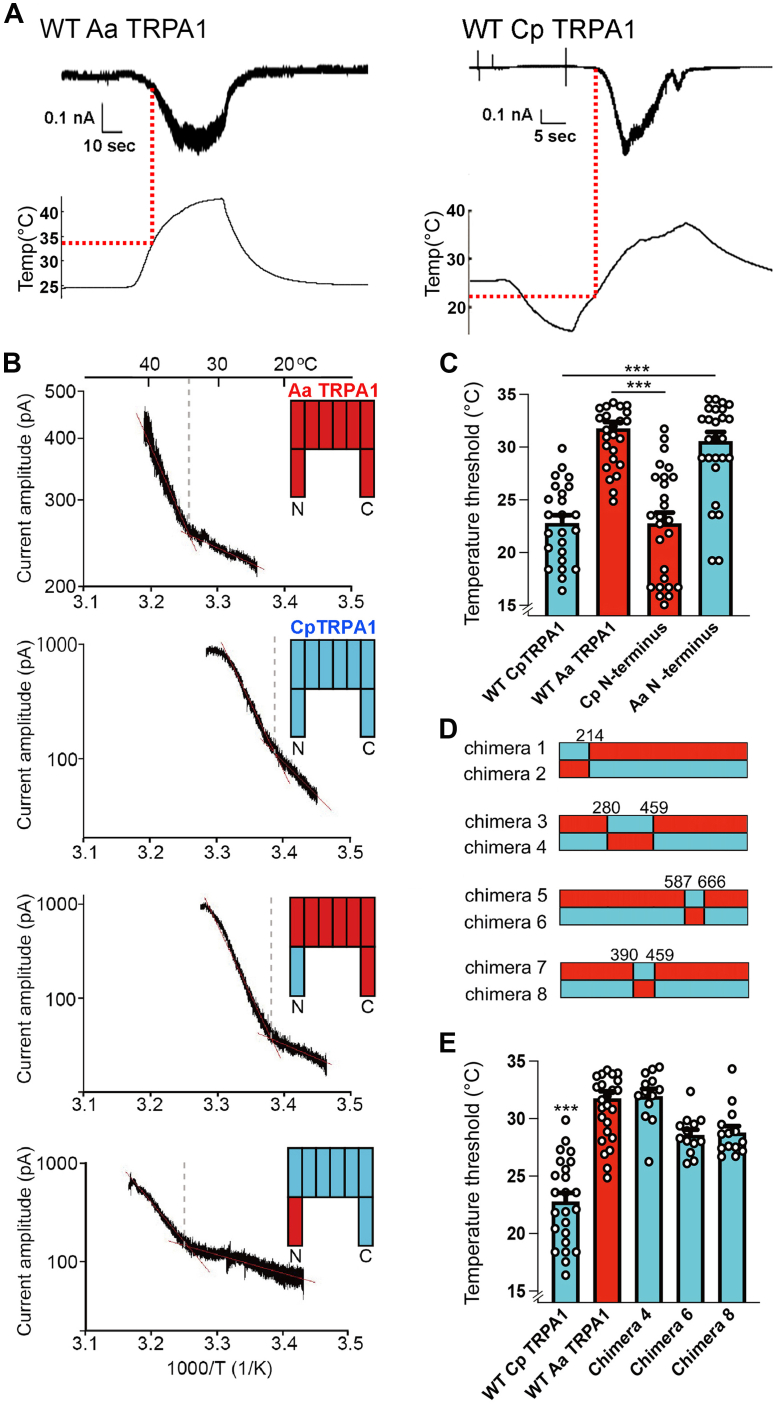
**Comparison of apparent temperature thresholds of heat-evoked activation WT TRPA1 from *Aede*s *aegypti* and *Culex pipiens pallens* with chimeras.***A*, representative current and temperature traces for WT TRPA1 from *Aede*s *aegypti* (WT *Aa* TRPA1) and *Culex pipiens pallens* (WT *Cp* TRPA1). Intersections of *dot lines* indicate apparent temperature thresholds. *B*, representative Arrhenius plots of heat-evoked currents from WT *Aa* TRPA1 (*red*), WT *Cp* TRPA1 (*blue*), and chimeras in which the N-terminal ARs were exchanged. *C*, apparent thresholds of WT *Aa* TRPA1, WT *Cp* TRPA1, and chimeras. Data presented as mean ± SEM with *circles* indicating individual data points. ∗∗∗*p* < 0.001. *D*, schematic of chimera design. Amino acids numbered from initial methionine; *red* and *blue* indicate *Aa* and *Cp*, respectively. *E*, apparent thresholds for chimeras in (*D*). Data presented as in (*C*). Chimeras 2, 3, 5, and 7 not shown because of negligible channel activity. ∗∗∗*p* < 0.001. Significance assessed by one-way ANOVA followed by a Bonferroni post hoc test or by Kruskal–Wallis ANOVA followed by Dunn's test for non-normally distributed datasets (*Aa*TRPA1 with *Cp* N terminus and Chimera 8). AR, ankyrin repeat; TRPA1, transient receptor potential ankyrin 1.

Because of their reported importance (6, 7, 8), we tested whether the ARs contained the threshold-determining regions for heat-evoked activation. We made chimeras by exchanging the AR domains of *Aa* and *Cp* TRPA1 channels, then expressed channels in human embryonic kidney–derived 293T (HEK293T) cells, and measured heat-evoked currents together with bath temperatures using a whole-cell patch-clamp method (see the Experimental procedures section). AR domain exchange was sufficient to exchange apparent channel thresholds: (WT *Cp* TRPA1: 22.8 ± 0.8 °C, n = 24; WT *Aa* TRPA1: 31.7 ± 0.6 °C, n = 29; *Cp* ARs in *Aa* TRPA1 (*Cp* N terminus): 22.8 ± 1.0 °C, n = 26; *Aa* ARs in *Cp* TRPA1 (*Aa* N terminus): 30.6 ± 0.9 °C, n = 29) (Figs. 1, *A*–*C*, S1 and Table S1). Such heat-evoked currents were never observed in the mock-transfected cells (n = 5; Fig. S2).

To determine a minimal region, we identified residues in the ARs whose chemical properties differed between *Aa* and *Cp* TRPA1. We generated chimeras by exchanging regions in which these residues clustered: ARs 2 to 6 in chimeras 1 and 2, ARs 7 to 12 in chimeras 3 and 4, and ARs 15 to 18 in chimeras 5 and 6 (Fig. 1*D*). Although chimeras 1, 2, 3, and 5 had negligible responses to heat and citronellal stimuli, chimeras 4 and 6, in which most of the ARs were from *Cp* TRPA1, had apparent thresholds similar to WT *Aa* TRPA1 (chimera 4: 31.65 ± 0.2 °C, n = 13; chimera 6: 28.6 ± 0.5 °C, n = 13) (Figs. 1*E*, S1 and Table S1), indicating that ARs 7 to 12 and 15 to 18 participate in threshold determination. Both chimeras had citronellal sensitivity comparable to WT *Cp* TRPA1 (Table S1, Figs. S3 and S4).

Because the apparent threshold of chimera 4 was comparable to WT *Aa* TRPA1, we generated chimeras 7 and 8 by exchanging ARs 11 to 12 (Fig. 1*D*). Although chimera 7 had negligible currents, AR 11 to 12 exchange was sufficient to raise the threshold of chimera 8 to 28.8 ± 0.2 °C (n = 14, Figs. 1*E*, S1 and Table S1).

**Figure 2:**
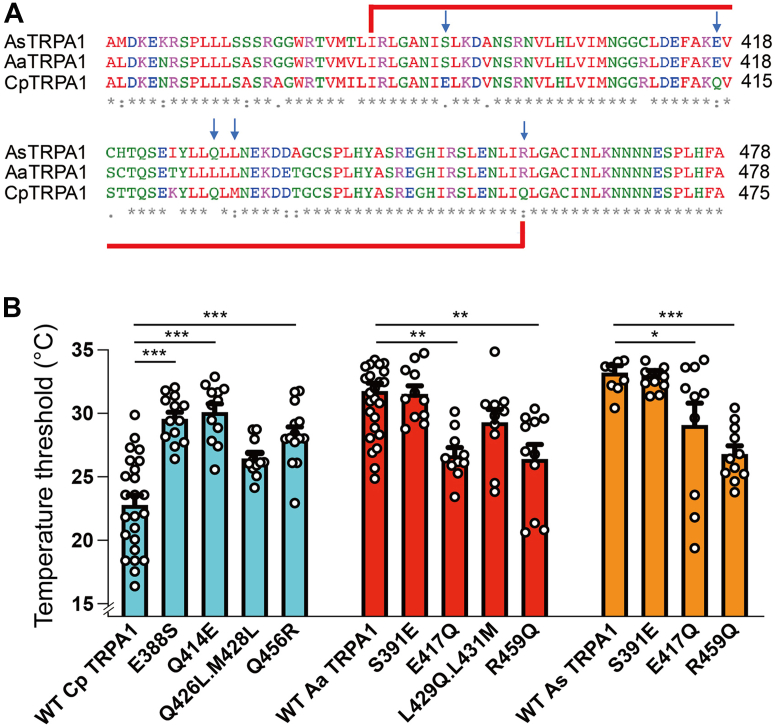
**Comparison of apparent temperature thresholds of heat-evoked activation of *Aa*, *Cp*, and *As* TRPA1 with point mutants**. *A*, alignment of WT *Aa* TRPA1, *Cp* TRPA1, and *As* TRPA1 with the region exchanged in chimera 8 bracketed in *red*. *Blue arrows* indicate point mutation sites. *B*, apparent activation thresholds for point mutants from (*A*). Data presented as mean ± SEM with *circles* indicating individual data points. ∗*p* < 0.05, ∗∗*p* < 0.01, and ∗∗∗*p* < 0.001. Significance assessed by one-way ANOVA followed by a Bonferroni post hoc test or by Kruskal–Wallis ANOVA followed by Dunn’s test for non-normally distributed datasets (*Aa*TRPA1 R459Q and *As*TRP1 E417Q). *Aa*, *Aedes aegypti*; *As*, *Anopheles stephensi*; *Cp*, *Culex pipiens pallens*; TRPA1, transient receptor potential ankyrin 1.

We sought to identify the crucial amino acids among the 70 exchanged in chimera 8 by focusing on positions, which differed in charge or polarity between *Cp* TRPA1 and *Aa* TRPA1. Finding five such positions (Fig. 2*A*), we mutated each to its counterpart in the other species in a series of single or double mutants: S391E, E417Q, L429Q.L431M, and R459Q in *Aa* TRPA1; E388S, Q414E, Q426L.M428L, and Q456R in *Cp* TRPA1 (Fig. 2, *A* and *B*). Apparent thresholds for *Cp* TRPA1 Q414E (30.1 ± 0.7 °C; n = 12) and Q456R (28.3 ± 0.6 °C; n = 14) were significantly higher than WT *Cp* TRPA1, and apparent thresholds for the corresponding *Aa* TRPA1 mutants E417Q (26.7 ± 0.6 °C; n =10) and R459Q (26.4 ± 1.1 °C; n = 11) were significantly lower than WT *Aa* TRPA1 (Figs. 2*B*, S5 and Table S1). The apparent threshold of *Cp* TRPA1 E388S increased relative to WT (29.6 ± 0.5 °C; n = 13, Fig. 2*B*), but the apparent threshold for the corresponding *Aa* TRPA1 mutant (S391E, 31.5 ± 0.6 °C; n = 11), along with those for the *Aa* TRPA1 L429Q.L431M and *Cp* TRPA1 Q426L.M428L mutants, did not differ significantly from their WT counterparts (Fig. S5 and Table S1). These results indicate that residues E417 and R459 of *Aa* TRPA1 and the corresponding residues of *Cp* TRPA1, along with E388, are important in threshold determination.

*Aa* TRPA1 E417 and R459 are conserved in TRPA1 of another tropical mosquito, *Anopheles stephensi* (*As* TRPA1) (Fig. 2*A*), which has an apparent threshold of 33.2 ± 0.5 °C (n = 9, Fig. 2*B*). To test if the roles of E417 and R459 were conserved, we generated E417Q and R459Q mutants of *As* TRPA1 and found that their apparent thresholds (E417Q: 29.1 ± 1.7 °C; n = 10; R459Q: 26.8 ± 0.6 °C; n = 11) decreased significantly relative to WT (Figs. 2*B*, S5 and Table S1). The *As* TRPA1 S391E threshold (33.0 ± 0.4 °C, n = 11) was comparable to WT, suggesting that the role of E338 is specific to *Cp* TRPA1 (Fig. 2*B* and Table S1). All mutants had unchanged citronellal sensitivities (Table S1, Fig. S3, Figs. S3 and S4).

To confirm that the currents seen upon heat stimulation of WT, chimeras, and mutants were heat evoked, we quantified Q_10_ values for all functional channels. For all channels, Q10 values were between 1.3 and 2.3 at temperatures below the apparent temperature thresholds of the channel in question and became greater (4–20) above the temperature thresholds (Table S2), indicating that observed currents were temperature dependent and thus represent true heat-evoked responses.

## Discussion

Sensing potentially harmful temperatures is crucial for survival, but what constitutes a harmful temperature varies depending on species and environment. Species that have evolved high temperature tolerance will not sustain damage at temperatures dangerous for species without such adaptations; therefore, species adapted to higher heat will need to recognize higher temperatures as noxious. In mosquitoes, which have adapted to environments from tropical to temperate, TRPA1 has been reported to be critical for detecting nociceptive temperatures (4). Indeed, differences in TRPA1 temperature thresholds reflect the difference in noxious temperature avoidance behaviors of the tropical *Aa* and the temperate Cp (12).

Here, we sought to determine the molecular basis of this thermosensory adaptation by identifying the structural features involved in threshold determination. We found that exchange of mosquito TRPA1 N-terminal AR domains is sufficient to exchange apparent temperature thresholds, consistent with reports on other TRPA1 homologs (6, 8). We narrowed down the region of interest to ARs 7 to 12 and 15 to 18 and further found that a single exchange of amino acid identity at certain positions is sufficient to alter apparent activation temperature thresholds by 5 to 7 °C. Notably, and unusually for cross-species mutations in TRP channels, amino acid exchanges in both directions—*Aa* to *Cp* and vice versa—resulted in apparent threshold-shifted functional channels, indicating that the role of these positions is likely conserved.

For mutations at positions important in all three species, namely *Aa* E417Q and R459Q and their counterparts, charged residues were associated with higher activation temperatures and polar residues with lower activation temperatures. The presence of conserved oppositely charged residues on helices adjacent to both E417 and R459 suggests that they may participate in salt bridge networks, although the lack of a solved TRPA1 AR structure prevents definitive conclusions (9). Thermodynamic analyses of TRP thermosensation suggest that temperature activation involves breakage of intermolecular interactions (13, 14), and studies of thermosensitive TRPV channels indicate that AR flexibility increases with temperature, potentially helping trigger channel opening (15, 16). If heat triggers opening in part by disrupting intermolecular interactions and thus increasing AR flexibility above a threshold, the threshold temperature is expected to increase with the strength of those interactions.

AR flexibility as key to temperature-evoked activation is consistent with the observed 5 to 7 °C apparent threshold changes caused by single-point mutations. In a study on designed AR proteins, a single-point mutation was observed to increase stability by 8 to 16 °C; molecular dynamics indicated that the mechanism was increased rigidly and decreased thermal fluctuation of the mutants (17). Even in the absence of a full AR structure for TRPA1, our results suggest an avenue for investigations of TRPA1 thermosensing mechanisms by targeted mutagenesis of residues likely to contribute to AR rigidity.

The 5 to 7 °C apparent threshold change elicited by single-point mutations also helps shed light on the adaptive evolution of TRPA1 channels. Single-point mutations in mouse TRPA1 ARs are reported to change activation stimulus from cold to heat, raising speculation that such plasticity allowed diversification of an ancestral TRPA1 into homologs responsive to different stimuli (8). Analogously, our results suggest a mechanism by which heat-responsive TRPA1 could evolve into orthologs adapted to the wide range of climates now inhabited by mosquitoes.

Finally, the threshold-setting amino acid positions we identify could provide a valuable insect repellent target, especially because while the role of ARs as a whole in temperature sensation may be common across homologs (6, 8), both sequence and temperature sensitivity function differ heavily between insect and mammalian TRPA1s, suggesting potential insect specificity. Targeting these positions may cause mosquitoes to misidentify human body temperature as noxious. Since the role of the positions we identified is conserved across at least three species from different habitats, our results suggest a possible avenue to counter mosquito-borne diseases across these many climates.

## Experimental procedures

### Construction of mosquito TRPA1 vector for mammalian expression

For WT TRPA1, we used *TRPA1* isoform A from each species cloned into the pcDNA3.1 vector as reported in our previous work (12). Isoform A was chosen because we had previously found that isoform A of TRPA1 for both *As* and Cp had the greatest apparent temperature-evoked current density (12).

### Construction of mutant mosquito TRPA1

*As TRPA1*, *Aa TRPA1*, or *Cp TRPA1* mutants were constructed using a PrimeSTAR mutagenesis Basal kit according to the manufacturer’s recommendations (Takara Bio, Inc). Point mutations were introduced by PCR using *Aa TRPA1*-pcDNA 3.1, *Cp TRPA1*-pcDNA3.1 or *As TRPA1*-pcDNA3.1 as templates with oligonucleotide primers (mentioned below) containing the intended mutations.

**Table:** 

Channel	Mutation	Sense primer (5’->3′)	Antisense primer (5’->3′)
*Aa* TRPA1	S391E	CAACATAGAATTGAAGGACGTAAATTC	CCTTCAATTCTATGTTGGCTCCGAGAC
	E417Q	CGCAAAACAGGTCAGTTGCACCCAATC	ACTGACCTGTTTTGCGAATTCATCCAAC
	L429Q/L431M	CCTTTTACAGCTGATGAATGAAAAGGATG	TTCATTCATCAGCTGTAAAAGGTATGT
	R456Q	TTGATACAGCTTGGAGCATGTATTAAC	CTCCAAGCTGTATCAAATTTTCGAGCG
*Cp* TRPA1	E388S	CAACATAAGCCTGAAGGATGTCAACTC	CCTTCAGGCTTATGTTGGCCCCTAAA
	Q414E	GCCAAGGAGGTGTCCACCACCCAATCG	GGACACCTCCTTGGCGAACTCATCCAAC
	Q426L/M428L	CTCTTACTGTTGCTGAACGAGAAGGACG	CTCGTTCAGCAACAGTAAGAGATACTTT
	Q456R	CTTATCCGCTTGGGTGCCTGCATCAACC	CACCCAAGCGGATAAGATTCTCCAGTG
*As* TRPA1	S391E	CAACATTGAACTGAAGGACGCCAATTC	CCTTCAATTCTATGTTGGCTCCGAGAC
	E417Q	CGCGAAGCAAGTGTGCCACACGCAGTC	GGCACACTTGCTTCGCGAACTCGTCCAA
	R459R	CGAGAACCTGATCCAGCTTGGGGCGTGC	GCACGCCCCAAGCTGGATCAGGTTCTCG

The amplified PCR products were directly transformed into *Escherichia coli* and subsequently purified using standard procedures. The entire mosquito *TRPA1* coding sequences were sequenced to confirm that only the intended mutations were introduced.

Chimeras were made through DNA assembly using a NEBuilder HiFi DNA Assembly Master Mix (New England Biolabs Japan, Inc) with fragments amplified by PCR from WT plasmid DNA and pcDNA3.1. The oligonucleotide primers used are shown here. The entire mosquito *TRPA1* coding sequences were determined to confirm that only the intended mutations were introduced.

**Table:** 

Chimera	Fragment	Template	Sense primer (5'->3′)	Antisense primer (5'->3′)
1	1-1-*Aa*	*Aa* TRPA1-A	TCTGGCTAACTAGAGAACCCACTGC	GATAATAGCCATTGTTGCAC GGCCGCCGAGGACACGCACC
	1-2-*Cp*	*Cp* TRPA1-A	GTGCGTGTCCTCGGCGG CCGTGCAACAATGGCTATTATCC	CTAGAAGGCACAGTCGAGGCTG
2	2-1-*Cp*	*Cp* TRPA1-A	TCTGGCTAACTAGAGAACCCACTGC	GGATAGTAACCGTTGTTGCAGGG TTTCCGCGGCGAAGCTC
	2-2-*Aa*	*Aa* TRPA1-A	GTTTGGAGCTTCGCCGCGGAAA CCCTGCAACAACGGTTAC	CTAGAAGGCACAGTCGAGGCTG
3	3-1-*Aa*	*Aa* TRPA1-A	TCTGGCTAACTAGAGAACCCACTGC	CCGACTTCAT GCAAAGCTCGACCGCTTTAATATCTCCACC
	3-2-*Cp*	*Cp* TRPA1-A	TCATGGTGGAGATATTAAA GCGGTCGAGCTTTGCATGAAG	AATACATGCTCCAAG CTGGATAAGATTCTCCAGTGAACGG
	3-3-*Aa*	*Aa* TRPA1-A	CACTGGAGAATCTTATCCAG CTTGGAGCATGTATTAACTT	CTAGAAGGCACAGTCGAGGCTG
4	4-1-*Cp*	*Cp* TRPA1-A	TCTGGCTAACTAGAGAACCCACTGC	GATTTTCGCTCCCGACTTTAG GCAAAGCTCGACCGCCTTG
	4-2-*Aa*	*Aa* TRPA1-A	GTGCACGGGGGCGATATCAAG GCGGTCGAGCTTTGC	AGGTTGATGCAGGCACCCAA GCGTATCAAATTTTCGAGCG
	4-3-*Cp*	*Cp* TRPA1-A	CGCTCGAAAATTTGATACGC TTGGGTGCCTGCATCAACCT	CTAGAAGGCACAGTCGAGGCTG
5	5-1-*Aa*	*Aa* TRPA1-A	TCTGGCTAACTAGAGAACCCACTGC	TCAGTAGCAGAACCACGGCAC TTGGTTTGTTTTCCATCGT
	5-2-*Cp*	*Cp* TRPA1-A	GCAACGATGGAAAACAAACCA AGTGCCGTGGTTCTGCTAC	AGTCCTTTTTGCAGTTCGCT TTCGTAATGCAATTGTCCTG
	5-3-*Aa*	*Aa* TRPA1-A	TCGAGGCCGTCCAGGAC AATTGCATTACGAAAGCGAACTG	CTAGAAGGCACAGTCGAGGCTG
6	6-1-*Cp*	*Cp* TRPA1-A	TCTGGCTAACTAGAGAACCCACTGC	AGCAAAAGAACCACAGCAT TAGGTCTGTTCTCCATCGTTG
	6-2-*Aa*	*Aa* TRPA1-A	AACGATGGAGAACAGACCT AATGCTGTGGTTCTTTTGCTG	AATCCTTCTTGCAGTTTGCC TTCGAAATGCAATTATCTTG
	6-3-*Cp*	*Cp* TRPA1-A	TGTCCAAGATAATTGCATTT CGAAGGCAAACTGCAAGAAG	CTAGAAGGCACAGTCGAGGCTG
7	7-1-*Aa*	*Aa* TRPA1-A	TCTGGCTAACTAGAGAACCCACTGC	GAGTTGACATCCTTCAGTTC TATGTTGGCTCCGAGAC
	7-2-*Cp*	*Cp* TRPA1-A	ATGGTTTTGATCCGTCTCGGA GCCAACATAGAACTGAAGG	AAGTTAATACATGCTCCAAG CTGGATAAGATTCTCCAGTG
	7-3-*Aa*	*Aa* TRPA1-A	CACTGGAGAATCTTATCCAG CTTGGAGCATGTATTAACTT	CTAGAAGGCACAGTCGAGGCTG
8	8-1-*Cp*	*Cp* TRPA1-A	TCTGGCTAACTAGAGAACCCACTGC	TTACGTCCTTCAAGCT TATGTTGGCCCCTAAACGAATGAG
	8-2-*Aa*	*Aa* TRPA1-A	ATTCTCATTCGTTTAGGG GCCAACATAAGCTTGAAGGAC	AGGTTGATGCAGGCACCCAA GCGTATCAAATTTTCGAGCG
	8-3-*Cp*	*Cp* TRPA1-A	CGCTCGAAAATTTGATACGC TTGGGTGCCTGCATCAACCT	CTAGAAGGCACAGTCGAGGCTG

### Cell culture

HEK293T cells were maintained at 37 °C and 5% CO_2_ in Dulbecco’s modified Eagle’s medium (WAKO Pure Chemical Industries) containing 10% fetal bovine serum (Biowest SAS), 100 units ml^−1^ penicillin (Invitrogen Corp), 100 μg ml^−1^ streptomycin (Invitrogen Corp), and 2 mM GlutaMAX (Invitrogen Corp). The cell line has not been authenticated.

For patch-clamp recordings, HEK293T cells cultured in 35 mm dishes were transfected with 1 μg *Aa TRPA1*, *Cp TRPA1*, or *As TRPA1* in pcDNA3 vector and 0.1 μg pGreen Lantern 1 complementary DNA using Lipofectamine Plus Reagent (Invitrogen Corp). After incubating for 3 to 4 h, the cells were reseeded on coverslips and further incubated at 33 °C for *Aa* TRPA1, *Cp* TRPA1, or *As* TRPA1 in 5% CO_2_. Patch-clamp recordings were performed 1 day after transfection.

### Chemicals

Citronellal was purchased from Sigma–Aldrich and diluted to the desired final concentration with bath solution.

### Electrophysiology

For whole-cell experiments, bath solution was 140 mM NaCl, 5 mM KCl, 2 mM MgCl_2_, 5 mM EGTA, 10 mM Hepes, and 10 mM glucose at pH 7.4 (adjusted with NaOH), and pipette solution was 140 mM KCl, 5 mM EGTA, and 10 mM Hepes at pH 7.4 (adjusted with KOH). Data from whole-cell voltage-clamp recordings were acquired at 10 kHz and filtered at 5 kHz for analysis (Axon 200B amplifier with pCLAMP software; Axon Instruments). Membrane potential was clamped at −60 mV. Series resistance and membrane capacitance were compensated.

All experiments were performed at 25 °C unless otherwise stated. To carry out heat stimulation, bath temperature was increased using a preheated solution warmed in an inline heater (1 °C s^−1^, with a maximum of 55 °C). Temperature was monitored using a thermocouple (TC-344; Warner Instruments) placed within 100 μm of the patch-clamped cell. Heat stimulation was stopped upon confirming that *Aa*TRPA1, *Cp*TRPA1, or *As*TRPA1 currents were desensitized or inactivated.

Temperature profiles and Arrhenius plots for the data from whole-cell voltage-clamp recordings were calculated using Origin software, version 9.0J (OriginLab). Absolute current values were plotted on a log scale against the reciprocal of the absolute temperature (*T*) (Arrhenius plot), and the apparent temperature threshold for channel activation was determined as the temperature at which a change of slope occurred. For current density analysis of channels, the peak currents induced by heat or chemical stimulation were measured and presented as pA pF^−1^. Q_10_ temperature coefficients were determined in the temperature range where the Arrhenius plot was linear (correlation coefficient >0.99) for each cell, using the formula Q_10_ = (I_2_/I_1_)^10/(T2 − T1)^, where I_1_ and I_2_ are the current amplitudes obtained at given temperatures T_1_ and T_2_ (18).

### Statistical analysis

Data for the patch-clamp experiments were obtained from at least three independent transfections. Data are presented as the mean ± SEM. Statistical analysis was performed with Origin software, version 9.0J. Normal distribution was confirmed for all datasets except *Aa*TRPA1 with *Cp*TRPA1 N terminus, Chimera 8, *Aa*TRPA1 R459Q, and *As*TRPA1 E417Q using the Shapiro–Wilk test. For the normally distributed datasets, significant changes were identified using one-way ANOVA followed by a Bonferroni post hoc test. For the four non-normally distributed datasets noted previously, significant changes were identified using the Kruskal–Wallis ANOVA followed by Dunn's test. For all datasets, *p* < 0.05 was considered as statistically significant (*p* values: ∗<0.05, ∗∗<0.01, and ∗∗∗<0.001).

## Data availability

All datasets are included within the article or as supporting information.

## Supporting information

This article contains supporting information.

## Conflict of interest

The authors declare that they have no conflicts of interest with the contents of this article.

## Supporting information

Nguyen et al. revised SI Figure 1

Nguyen et al. revised SI Figure 2

Nguyen et al. revised SI Figure 3

Nguyen et al. revised SI Figure 4

Nguyen et al. revised SI Figure 5

Nguyen et al. SI Table 1

Nguyen et al. revised SI Table 2
