# Supplementary material for: Single amino acids set apparent temperature thresholds for heat-evoked activation of mosquito transient receptor potential channel TRPA1
Source: J Biol Chem. 2022 Jul 16;298(9):102271. doi: 10.1016/j.jbc.2022.102271 (PMC9396403; doi:10.1016/j.jbc.2022.102271)
Supplement: Nguyen et al. revised SI Figure 1 [file mmc1.pdf]

WT Cp TRPA1

Heat-evoked current
Threshold

Data#	℃
1	21.90381028
2	24.91172783
3	20.42809801
4	16.39541162
5	25.58276158
6	27.31343932
7	26.2902907
8	22.11024603
9	27.1503003
10	26.2511976
11	29.88030303
12	28.05481928
13	23.58590504
14	25.04767442
15	20.06428571
16	18.3951895
17	23.58590504
18	18.3951895
19	23.58590504
20	17.54767442
21	20.96764706
22	18.3951895
23	19.24766082
24	21.83525074
**Average**	**22.83002746**
**S.D.**	**3.720373095**
**S.E.**	**0.759417978**

3mM Citronellal
Current density

Data#	pA/pF
1	62.29306488
2	67.78151261
3	187.8386763
4	37.32121212
5	47.92592593
6	11.84
**Average**	69.16673197
**S.D.**	61.45544676
**S.E.**	25.08908108

Heat-evoked current
Current density

Data#	pA/pF
1	68.07874016
2	65.15151515
3	189.9357798
4	61.37142857
5	42.93537788
6	166.2691131
7	31.43
8	66.65536723
9	45.23243243
10	33.07692308
11	42.62820513
12	39.96747967
13	21.20089286
14	45.53051643
15	189.0410959
16	72.64130435
17	76.94623656
18	12.61344538
19	170.8981481
20	458.4274194
21	47.14765101
22	47.33333333
23	8.473214286
24	85.5
25	9.288888889
26	166.1818182
27	32.85714286
28	41.91089109
29	4.925675676
**Average**	**80.8155185**
**S.D.**	**91.06089173**
**S.E.**	**16.90958308**

WT Aa TRPA1

Heat-evoked current
Threshold

Data#	℃
1	24.46904762
2	29.88030303
3	37.40900621
4	25.35746269
5	34.54230769
6	27.1503003
7	33.59846626
8	28.05481928
9	32.66039755
10	33.59846626
11	30.80136778
12	30.80136778
13	36.44752322
14	28.87355784
15	28.32723847
16	27.24050766
17	30.80136778
18	31.63512649
19	35.01640986
20	32.47347188
21	35.77801977
22	33.88101013
23	32.47347188
24	32.94121518
25	29.69675954
26	36.16023817
27	33.69258975
28	33.31644192
29	32.00715594
**Average**	**31.69260062**
**S.D.**	**3.31951867**
**S.E.**	**0.616419142**

Heat-evoked current
Current density

Data#	pA/pF
1	9.091549296
2	5.585365854
3	17.82882883
4	5.160305344
5	17.6039604
6	19.31914894
7	3.455
8	21.25301205
9	13.19047619
10	12.51363636
11	4.264705882
12	9.644927536
13	52.664
14	7.403100775
15	11.52592593
16	8.3
17	25.92380952
18	24.78861789
19	16.32967033
20	12.35238095
21	38.5
22	4.088435374
23	3.847133758
24	4.202797203
25	24.00578035
26	8.39516129
27	4.493333333
**Average**	**14.28633568**
**S.D.**	**11.55320103**
**S.E.**	**2.223414575**

3mM Citronellal
Current density

Data#	pA/pF
1	1.224279835
2	7.17
3	3.985714286
4	6.175572519
5	5.375634518
6	11.52941176

Average **5.910102154**
S.D. **3.440357909**
S.E. **1.404520235**

WT As TRPA1

Heat-evoked current

Threshold

**Data#**	**℃**
1	33.70000631
2	30.42078321
3	32.60566355
4	33.66333375
5	36.14922375
6	34.16834665
7	32.21116645
8	32.00699484
9	34.05615631
**Average**	**33.22018609**
**S.D.**	**1.632022198**
**S.E.**	**0.544007399**

Heat-evoked current

Current density

**Data#**	**pA/pF**
1	10.225
2	12.06299213
3	27.26168224
4	20.6283857
5	20.625
6	10.11643836
7	95.81818182
8	22.77272727
9	37.71458118
**Average**	**28.5805543**
**S.D.**	**26.73248707**
**S.E.**	**8.910829024**

3mM Citronellal

Current density

**Data#**	**pA/pF**
1	11.78125
2	4.287292818
3	2.97029703
4	115.3153153
5	17.56521739
6	3.443548387

**Average**	**25.89382016**
**S.D.**	**44.18388551**
**S.E.**	**18.03799573**

Cp N-terminus

Heat-evoked current
Threshold

Data#	℃
1	21.21627141
2	27.15791171
3	23.40965797
4	26.4786256
5	28.38393358
6	25.16186894
7	23.52960899
8	23.05275854
9	17.54767442
10	18.3951895
11	16.70507246
12	16.70507246
13	30.80136778
14	15.03443804
15	15.86734104
16	24.46904762
17	27.1503003
18	27.1503003
19	16.70507246
20	16.70507246
21	22.70798817
22	28.05481928
23	15.86734104
24	21.83525074
25	31.72804878
26	29.88030303
**Average**	**22.75770525**
**S.D.**	**5.245082402**
**S.E.**	**1.028645289**

Heat-evoked current
Current density

Data#	pA/pF
1	23.42857143
2	12.29770992
3	10.17283951
4	21.15533981
5	18.7388535
6	16.03092784
7	11.35333333
8	15.17142857
9	31.45299145
10	13.34759358
11	23.41818182
12	23.95833333
13	18.54205607
14	5.503184713
15	3.687116564
16	12.38518519
17	40.91262136
18	28.56923077
19	15.90410959
20	21.97979798
21	145.6847826
22	83.16949153
23	80.59195402
24	31.86
25	84.57692308
26	85.47244094
27	36.75706215
28	109.8413793
29	36.59649123
30	13.74736842
**Average**	**35.87690999**
**S.D.**	**34.59891913**
**S.E.**	**6.316869491**

3mM Citronellal
Current density

Data#	pA/pF
1	4.315789474
2	41.26351351
3	38.02208835
4	38.02985075
5	15.92436975
6	9.607843137

**Average**	24.5272425
**S.D.**	16.42945399
**S.E.**	**6.707296505**

Aa N-terminus

Heat-evoked current

Threshold

**Data#**	**℃**
1	32.76222891
2	33.67749316
3	34.04219313
4	35.21681028
5	32.37732695
6	34.24204336
7	32.61709457
8	30.73770469
9	32.66935329
10	33.6115535
11	37.91713921
12	28.96480363
13	28.96480363
14	32.66039755
15	23.58590504
16	23.58590504
17	34.54230769
18	28.96480363
19	19.24766082
20	28.96480363
21	33.59846626
22	28.96480363
23	30.80136778
24	30.80136778
25	34.54230769
26	19.24766082
27	36.44752322
28	28.05481928
29	24.46904762
**Average**	**30.56136882**
**S.D.**	**4.731528465**
**S.E.**	**0.878622778**

Heat-evoked current

Current density

**Data#**	**pA/pF**
1	7.450980392
2	9.843478261
3	13.35928144
4	45.6433121
5	28.62222222
6	35.22233713
7	38.94915254
8	18.68
9	18.27731092
10	46.86713287
11	14.16788321
12	34.23809524
13	8.019607843
14	13.28645833
15	83.78350515
16	29.53416149
17	85.85
18	19.49038462
19	98.1483871
20	43.19852941
21	92.3
22	9.5375
23	8.6375
24	43.74683544
25	68.89690722
26	23.63636364
27	46.072
28	86.98969072
29	26.64705882
30	18.0070922
31	13.51322751
32	206.0921053
**Average**	**41.77214066**
**S.D.**	**40.58973967**
**S.E.**	**7.175320042**

3mM Citronellal

Current density

**Data#**	**pA/pF**
1	26.42774566
2	61.71111111
3	45.40909091
4	12.47413793
5	79.36538462
6	9.182608696

**Average**	39.09501315
**S.D.**	28.06043794
**S.E.**	11.45562582

Chimera 4

Heat-evoked current

Threshold

**Data#**	**℃**
1	33.26683817
2	33.00419834
3	31.29843726
4	32.52975119
5	34.48961149
6	34.28360537
7	33.98148686
8	32.3625726
9	30.21969957
10	32.00542853
11	29.8937466
12	32.00542853
13	26.25510019
**Average**	**31.96891575**
**S.D.**	**2.225871226**
**S.E.**	**0.617345603**

Heat-evoked current

Current density

**Data#**	**pA/pF**
1	76.30630631
2	16.4
3	163.5976331
4	132.9090909
5	49.90756303
6	59.85436893
7	151.1558442
8	40.47540984
9	13.97241379
10	21.97037037
11	127.4
12	22.81981982
13	78.54615385
**Average**	**73.48576724**
**S.D.**	**53.60127245**
**S.E.**	**14.86631817**

3 mM citronellal

Current density

**Data#**	**pA/pF**		
1	146.4829932		
2	21.59090909		
3	412.7868852		
4	162.3636364	**Average**	**154.8249856**
5	118.0636943	**S.D.**	**136.6534999**
6	67.66179541	**S.E.**	**55.78855772**

Chimera 6

Heat-evoked current
Threshold

Data#	℃
1	32.36671559
2	29.50511016
3	29.86198335
4	26.10546648
5	28.94473667
6	28.28327362
7	28.01979365
8	27.84390627
9	29.83866214
10	28.06342919
11	26.31615866
12	29.37842463
13	27.0508094
**Average**	**28.58295922**
**S.D.**	**1.68301394**
**S.E.**	**0.466784081**

Heat-evoked current
Current density

Data#	pA/pF
1	75.62944162
2	149.5233266
3	234.224359
4	87.48577929
5	89.07615481
6	216.8443051
7	18.05128205
8	33.04347826
9	75.94690265
10	172.1609195
11	8.895833333
12	89.62251656
13	142.9895833
**Average**	**107.1918371**
**S.D.**	**71.62348376**
**S.E.**	**19.86478025**

3 mM citronellal
Current density

Data#	pA/pF		
1	211.2176871		
2	40.93877551		
3	67.84782609	**Average**	**104.1485101**
4	60.4025974	**S.D.**	**70.71109155**
5	140.3356643	**S.E.**	**31.62296149**

Chimera 8

Heat-evoked current

Threshold

**Data#**	**℃**
1	27.43267202
2	28.73476277
3	28.88950467
4	28.65302024
5	27.61394627
6	27.97665993
7	27.18042645
8	27.51522012
9	26.70149851
10	26.73114326
11	29.49081341
12	30.37551771
13	34.32754961
14	31.54307925
**Average**	**28.79755816**
**S.D.**	**2.111931433**
**S.E.**	**0.564437417**

Heat-evoked current

Current density

**Data#**	**pA/pF**
1	345.1851852
1	96.20833333
2	125.8125
3	87.72340426
4	63.26086957
5	74.16037736
6	182.6923077
7	88.5
8	111.0638298
9	114.5955056
10	42.21008403
11	88.01781737
12	35.72173913
13	151.5037037
**Average**	**114.7611184**
**S.D.**	**77.20472843**
**S.E.**	**20.6338316**

3mM citronellal

Current density

**Data#**	**pA/pF**		
1	49.21818182		
2	11.19166667		
3	38.11612903	**Average**	**34.86798609**
4	19.50406504	**S.D.**	**19.18887616**
5	56.30988787	**S.E.**	**8.581526302**

Cp TRPA1 E388S

Heat-evoked current

Threshold

**Data#**	**℃**
1	26.42083811
2	30.6153489
3	27.39404874
4	28.15832141
5	31.38858667
6	30.58689722
7	31.80309513
8	27.72132878
9	32.04478421
10	29.41056858
11	29.42938914
12	28.50499029
13	31.02825425
**Average**	**29.57741934**
**S.D.**	**1.823755147**
**S.E.**	**0.505818669**

Heat-evoked current

Current density

**Data#**	**pA/pF**
1	129.9919355
2	95.42622951
3	157.4468085
4	124.5165563
5	34.75373134
6	111
7	58.63358779
8	225.7422325
9	128.7185629
10	284.5780969
11	70.11009174
12	40.136
13	28.03636364
**Average**	**113.2581884**
**S.D.**	**78.77633842**
**S.E.**	**21.84862519**

3mM Citronellal

Current density

**Data#**	**pA/pF**
1	15.40384615
2	3.447154472
3	1.072
4	287.7446809
5	69.38596491
6	14.01851852

**Average**	**65.17869415**
**S.D.**	**111.8648022**
**S.E.**	**45.66861426**

Cp TRPA1 Q414E

Heat-evoked current

Threshold

**Data#**	**℃**
1	31.66681127
2	30.67626432
3	31.01273777
4	28.86035275
5	31.92087031
6	27.42239504
7	27.83793209
8	25.57214047
9	32.25657534
10	31.64268546
11	29.47595624
12	32.89371798
**Average**	**30.10320325**
**S.D.**	**2.266058997**
**S.E.**	**0.654154886**

Heat-evoked current

Current density

**Data#**	**pA/pF**
1	31.17647059
2	103.2113821
3	9.160305344
4	86.3553719
5	57.46478873
6	116.8833333
7	51.33056133
8	69.47058824
9	21.2962963
10	66.52
11	56.7890625
12	60.89147287
**Average**	**60.8791361**
**S.D.**	**31.51710798**
**S.E.**	**9.098205388**

3mM Citronellal

Current density

**Data#**	**pA/pF**
1	130.5508475
2	48.08333333
3	45.05185185
4	45.05660377
5	8.265486726
6	120.1568627

**Average**	**66.19416431**
**S.D.**	**48.23128283**
**S.E.**	**19.69033876**

Cp TRPA1 Q426L.M428L

Heat-evoked current
Threshold

**Data#**	**℃**
1	27.97964389
2	25.66969091
3	25.07090356
4	26.15937325
5	24.14516754
6	26.29902248
7	28.72767096
8	25.90513888
9	28.74929587
10	26.13367052
11	26.04695806
**Average**	**26.44423054**
**S.D.**	**1.459577735**
**S.E.**	**0.440079246**

Heat-evoked current
Current density

**Data#**	**pA/pF**
1	86.30411827
2	55.61682243
3	92.04878049
4	218.3146067
5	107.3412698
6	56.79398148
7	164.648
8	29.6796875
9	89.34166667
10	19.83333333
11	204.0088496
**Average**	**102.175556**
**S.D.**	**66.7608029**
**S.E.**	**20.12913945**

3mM Citronellal
Current density

**Data#**	**pA/pF**		
1	101.6747967		
2	355.4628422		
3	135.0508475	**Average**	**145.9754959**
4	29.109375	**S.D.**	**123.5141592**
5	108.5796178	**S.E.**	**55.23721122**

Cp TRPA1 Q456R

Heat-evoked current
Threshold

Data#	℃
1	26.10655365
2	31.77693563
3	29.18885681
4	31.67535674
5	28.02331129
6	22.93176992
7	28.38737777
8	26.09783309
9	27.98896508
10	28.06110207
11	28.02429272
12	27.752186
13	31.01273777
14	29.35511016
**Average**	**28.31302776**
**S.D.**	**2.350691712**
**S.E.**	**0.628248786**

Heat-evoked current
Current density

Data#	pA/pF
1	119.6371681
2	83.98076923
3	64.84397163
4	49.27868852
5	56.984
6	142.1785714
7	23.59243697
8	158.8402367
9	27.25694444
10	72.33333333
11	134.0107527
12	78.10344828
13	27.76296296
14	42.4952381
**Average**	**77.23560874**
**S.D.**	**45.01594424**
**S.E.**	**12.03101716**

3mM Citronellal
Current density

Data#	pA/pF		
1	106.3204748		
2	280.46		
3	72.63366337	**Average**	**161.3781517**
4	239.036965	**S.D.**	**92.08799364**
5	108.4396552	**S.E.**	**41.18300274**

Aa TRPA1 S391E

Heat-evoked current

Threshold

**Data#**	**℃**
1	29.73084143
2	29.17204402
3	33.41907633
4	31.14042223
5	31.62459267
6	31.02024555
7	33.0666084
8	34.7519387
9	28.78437913
10	29.86887738
11	34.37542169
**Average**	**31.54131341**
**S.D.**	**2.095732304**
**S.E.**	**0.631887065**

Heat-evoked current

Current density

**Data#**	**pA/pF**
1	13.81034483
2	51.15044248
3	16.78409091
4	7.551020408
5	9.050847458
6	20.50546448
7	25.32231405
8	19.56626506
9	15.98360656
10	18.77862595
11	42.46315789
**Average**	**19.85030222**
**S.D.**	**12.20216159**
**S.E.**	**3.679090148**

3mM Citronellal

Current density

**Data#**	**pA/pF**
1	0.4
2	2.284768212
3	1.924528302
4	1.506329114
5	2.661654135
6	4.460869565

**Average**	**2.206358221**
**S.D.**	**1.351196712**
**S.E.**	**0.55162374**

Aa TRPA1 E417Q

Heat-evoked current

Threshold

**Data#**	**℃**
1	26.73772863
2	26.17973039
3	29.08504113
4	27.80362724
5	26.4723108
6	26.10364674
7	25.89013976
8	23.4244084
9	30.1378606
10	25.27312386
**Average**	**26.71076175**
**S.D.**	**1.90977237**
**S.E.**	**0.60392305**

Heat-evoked current

Current density

**Data#**	**pA/pF**
1	35.56407448
2	21.93333333
3	12.84677419
4	34.06716418
5	40.12871287
6	30.22641509
7	12.76666667
8	12.90967742
9	29.99047619
**Average**	**25.60369938**
**S.D.**	**10.75038933**
**S.E.**	**3.583463111**

3mM Citronellal

Current density

**Data#**	**pA/pF**
1	4.317307692
2	1.37
3	1.214285714
4	0.76969697
5	12.7961165

**Average**	**4.093481376**
**S.D.**	**5.063117065**
**S.E.**	**2.264294787**

Aa TRPA1 L429Q.L431M

Heat-evoked current

Threshold

**Data#**	**℃**
1	29.82020292
2	30.92267244
3	30.68974962
4	23.82848006
5	30.18165936
6	29.13496266
7	28.36032461
8	34.8650525
9	24.50850952
10	30.71799065
**Average**	**29.30296044**
**S.D.**	**3.204145959**
**S.E.**	**1.013239919**

Heat-evoked current

Current density

**Data#**	**pA/pF**
1	28.52287582
2	13.13392857
3	21.98795181
4	38.0546875
5	33.84328358
6	11.73728814
7	8.876923077
8	14.83478261
9	7.669565217
10	10
**Average**	**18.86612863**
**S.D.**	**11.05139629**
**S.E.**	**3.494758361**

3mM Citronellal

Current density

**Data#**	**pA/pF**
1	4.360544218
2	4.169014085
3	4.946969697
4	1.161904762
5	2.207692308

**Average**	**3.369225014**
**S.D.**	**1.607255077**
**S.E.**	**0.718786322**

Aa TRPA1 R459Q

Heat-evoked current

Threshold

**Data#**	**℃**
1	29.59789062
2	20.63153938
3	27.0182443
4	29.66732793
5	30.37043611
6	29.34124896
7	25.94530281
8	28.96055674
9	26.78444423
10	20.81469031
11	21.3556626
**Average**	**26.40794036**
**S.D.**	**3.775260744**
**S.E.**	**1.138283943**

Heat-evoked current

Current density

**Data#**	**pA/pF**
1	16.67515924
2	9.56779661
3	26.48543689
4	13.95901639
5	34.00714286
6	13.15517241
7	19.42424242
8	16.08275862
9	15.13043478
10	5.806451613
11	12.69166667
**Average**	**16.63502532**
**S.D.**	**7.817898807**
**S.E.**	**2.357185181**

3mM Citronellal

Current density

**Data#**	**pA/pF**
1	1.574626866
2	3.313043478
3	13.00970874
4	1.275
5	3.967479675

**Average**	**4.627971751**
**S.D.**	**4.821414098**
**S.E.**	**2.236067977**

As TRPA1 S391E

Heat-evoked current

Threshold

**Data#**	**℃**
1	32.37773115
2	35.88704292
3	33.16759298
4	32.50656444
5	32.21116645
6	34.17447812
7	31.35096055
8	33.24528085
9	32.91652188
10	33.62514346
11	31.43375123
**Average**	**32.99056673**
**S.D.**	**1.28937511**
**S.E.**	**0.388761223**

Heat-evoked current

Current density

**Data#**	**pA/pF**
1	6.532190403
2	20.48277957
3	67.85568316
4	46.34352643
5	20.8679524
6	17.30984507
7	20.18385403
8	83.66205463
9	57.78672203
10	30.60495742
11	237.0952708
**Average**	**55.33862145**
**S.D.**	**64.93096512**
**S.E.**	**19.5774226**

3mM Citronellal

Current density

**Data#**	**pA/pF**
1	6.73015664
2	51.29714048
3	11.87505076
4	6.745550121
5	15.06254566

**Average**	**18.34208873**
**S.D.**	**18.76119728**
**S.E.**	**8.390262491**

As TRPA1 R459Q

Heat-evoked current

Threshold

**Data#**	**℃**
1	23.78792769
2	26.43103413
3	25.71027572
4	24.64713296
5	25.23265768
6	26.80780703
7	29.55574743
8	28.06110207
9	28.84308217
10	25.30685331
11	30.43673796
**Average**	**26.80185074**
**S.D.**	**1.878771691**
**S.E.**	**0.566470979**

Heat-evoked current

Current density

**Data#**	**pA/pF**
1	10.12280702
2	13.13559322
3	22.3
4	37.875
5	18.0234375
6	44.93700787
7	18.88405797
8	23.42038217
9	35.04464286
10	46.11561866
11	26.88148148
**Average**	**26.97636625**
**S.D.**	**13.03216236**
**S.E.**	**3.929344797**

3mM Citronallal

Current density

**Data#**	**pA/pF**
1	3.64
2	10.49723757
3	18.55855856
4	85.23573201
5	27.30263158
6	28.1328125

**Average**	**28.89449537**
**S.D.**	**29.19085253**
**S.E.**	**11.91711564**

As TRPA1 E417Q

Heat-evoked current

Threshold

**Data#**	**℃**
1	21.82141057
2	19.38963749
3	31.97676989
4	34.22967325
5	33.66333375
6	31.3359127
7	33.62870749
8	29.63756558
9	31.59193227
10	23.58247545
**Average**	**29.08574184**
**S.D.**	**5.427610648**
**S.E.**	**1.71636119**

Heat-evoked current

Current density

**Data#**	**pA/pF**
1	5.520547945
2	17.88793103
3	35.55555556
4	29.125
5	11.31818182
6	12.32894737
7	28.06542056
8	28.75862069
9	14.30128205
10	74.61538462
**Average**	**25.74768716**
**S.D.**	**19.72110068**
**S.E.**	**6.23635961**

3mM Citronellal

Current density

**Data#**	**pA/pF**
1	21.43011918
2	20.28776978
3	157.55
4	24.21296296
5	15.77702703

**Average**	**47.85157579**
**S.D.**	**61.39861916**
**S.E.**	**27.45829723**
