# Supplementary material for: Single amino acids set apparent temperature thresholds for heat-evoked activation of mosquito transient receptor potential channel TRPA1
Source: J Biol Chem. 2022 Jul 16;298(9):102271. doi: 10.1016/j.jbc.2022.102271 (PMC9396403; doi:10.1016/j.jbc.2022.102271)
Supplement: Nguyen et al. revised SI Figure 2 [file mmc2.pdf]

SI Table 2. Q10 values

	below thresholds	above thresholds
Cp TRPA1 WT (n = 24)	1.7 ± 0.13	9.96 ± 3.28
Aa TRPA1 WT (n = 25)	1.79 ± 0.18	8.1 ± 3.48
As TRPA1 WT (n = 9)	1.44 ± 0.05	7.99 ± 2.13
Cp N-term (n = 25)	1.53 ± 0.16	11.19 ± 4.82
Aa N-term (n = 29)	1.32 ± 0.06	11.54 ± 3.83
Chimera 4 (n = 13)	1.53 ± 0.27	13.89 ± 3.61
Chimera 6 (n = 13)	1.52 ± 0.19	13.16 ± 2.53
Chimera 8 (n = 14)	1.86 ± 0.17	10.9 ± 1.18
CpTRPA1 E388S (n = 13)	1.81 ± 0.16	20.53 ± 3.77
CpTRPA1 Q414E (n = 12)	2.27 ± 0.46	8.88 ± 1.46
CpTRPA1 Q426L.M428L (n = 11)	2.26 ± 0.24	8.78 ± 1.72
CpTRPA1 Q456R (n = 14)	1.66 ± 0.08	9.18 ± 1.38
AaTRPA1 S391E (n = 11)	1.49 ± 0.11	6.33 ± 1.46
AaTRPA1 E417Q (n = 10)	1.56 ± 0.35	5.28 ± 0.72
AaTRPA1 L429Q.L431M (n = 10)	1.29 ± 0.1	8.08 ± 2.27
AaTRPA1 R459Q (n = 11)	1.75 ± 0.14	4 ± 0.72
AsTRPA1 S391E (n = 11)	1.4 ± 0.08	10.52 ± 2.66
AsTRPA1 E417Q (n = 10)	1.44 ± 0.09	6.53 ± 1.42
AsTRPA1 R459Q (n = 11)	1.39 ± 0.1	8.42 ± 2.22
