# Supplementary material for: Single amino acids set apparent temperature thresholds for heat-evoked activation of mosquito transient receptor potential channel TRPA1
Source: J Biol Chem. 2022 Jul 16;298(9):102271. doi: 10.1016/j.jbc.2022.102271 (PMC9396403; doi:10.1016/j.jbc.2022.102271)

SI Figure 1. Representative current and temperature traces, and Arrhenius plots for wild type (WT) and chimeras of mosquito TRPA1. Aa: *Aedes aegypti*, Cp: *Culex pipiens pallens*, As: *Anopheles stephensi*. Below each current trace, the corresponding temperature trace is shown. Each Arrhenius plot is derived from the corresponding current trace.

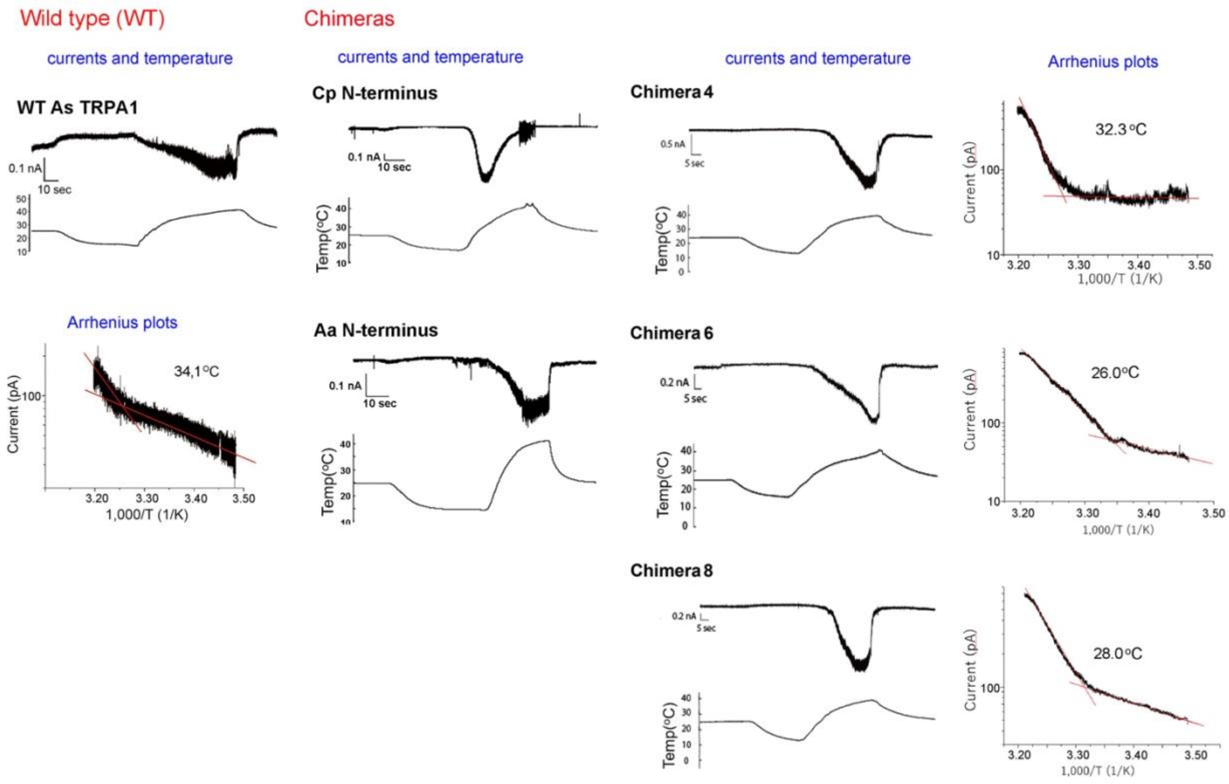

Supplement: Nguyen et al. revised SI Figure 3 [file mmc3.pdf]
