# Supplementary material for: Single amino acids set apparent temperature thresholds for heat-evoked activation of mosquito transient receptor potential channel TRPA1
Source: J Biol Chem. 2022 Jul 16;298(9):102271. doi: 10.1016/j.jbc.2022.102271 (PMC9396403; doi:10.1016/j.jbc.2022.102271)

SI Figure 2. A representative response to the heat stimulation in the mock-transfected cell. Similar responses were observed in other 4 cells.

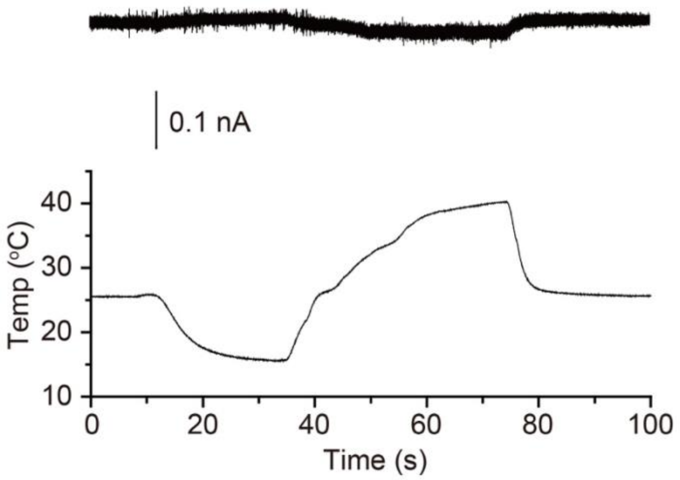

Supplement: Nguyen et al. revised SI Figure 4 [file mmc4.pdf]
