# Supplementary material for: Single amino acids set apparent temperature thresholds for heat-evoked activation of mosquito transient receptor potential channel TRPA1
Source: J Biol Chem. 2022 Jul 16;298(9):102271. doi: 10.1016/j.jbc.2022.102271 (PMC9396403; doi:10.1016/j.jbc.2022.102271)

SI Figure 3. Comparison for densities of the citronellal (3 mM)-evoked and heat-evoked currents in WT, chimeras and point mutants of mosquito TRPA1.

A. Densities of the citronellal-evoked currents in WT and chimeras (n = 13-29). **B.** Densities of the citronellal-evoked currents in point mutants (n = 10-14). **C.** Densities of the heat-evoked currents in WT and chimeras (n = 13-29). **D.** Densities of the heat-evoked currents in point mutants (n = 10-14).

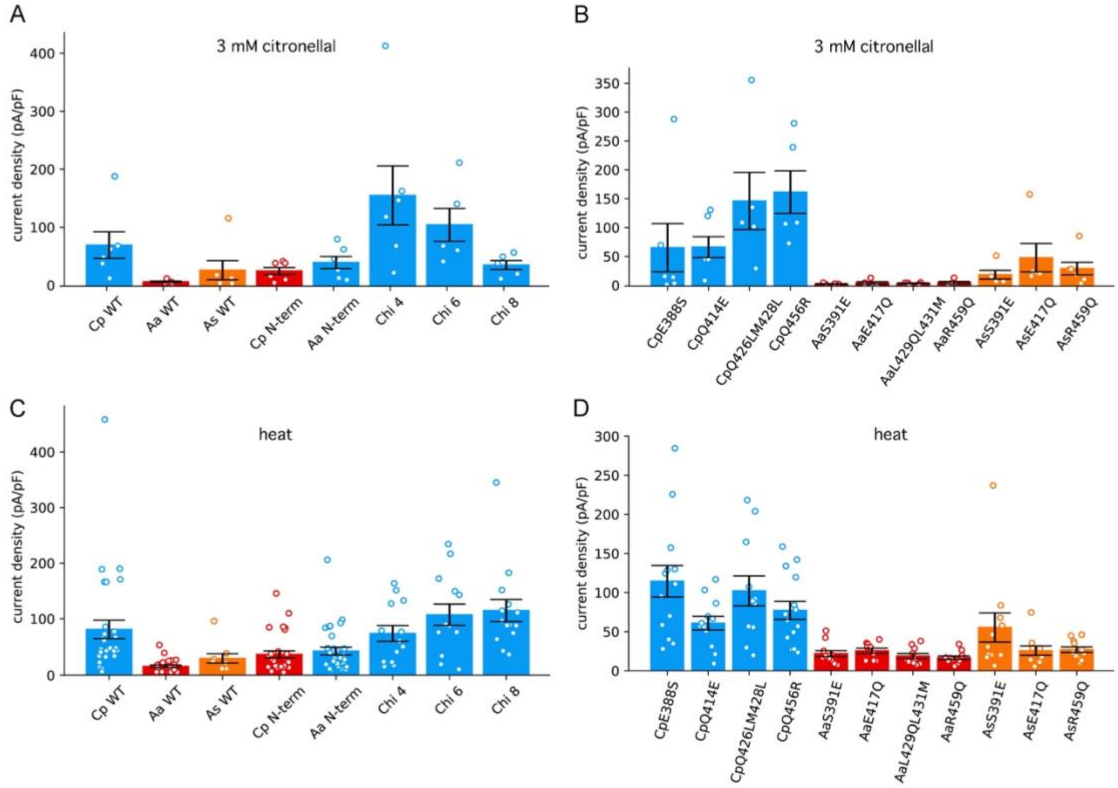

Supplement: Nguyen et al. revised SI Figure 5 [file mmc5.pdf]
