# Supplementary material for: Single amino acids set apparent temperature thresholds for heat-evoked activation of mosquito transient receptor potential channel TRPA1
Source: J Biol Chem. 2022 Jul 16;298(9):102271. doi: 10.1016/j.jbc.2022.102271 (PMC9396403; doi:10.1016/j.jbc.2022.102271)

SI Figure 4. Representative citronellal (3 mM)-evoked current traces and dose dependency of mosquito TRPA1.

A. Representative citronellal-evoked current traces of WT Cp TRPA1 and Cp TRPA1 mutant Q414E. **B.** Citronellal dose-dependency for the currents of WT Cp TRPA1 and Cp TRPA1 mutant Q414E. Box-and-whisker plots with mean (open circles) and individual data (closed circles) (n = 6-8). **C.** Representative citronellal-evoked current traces of WT Aa TRPA1 and Aa TRPA1 mutant E417Q. NS: no significant. **D.** Citronellal dose-dependency for the currents of WT Aa TRPA1 and Aa TRPA1 mutant E417Q. Box-and-whisker plots with mean (open circles) and individual data (closed circles) (n = 6-7). NS: no significant.

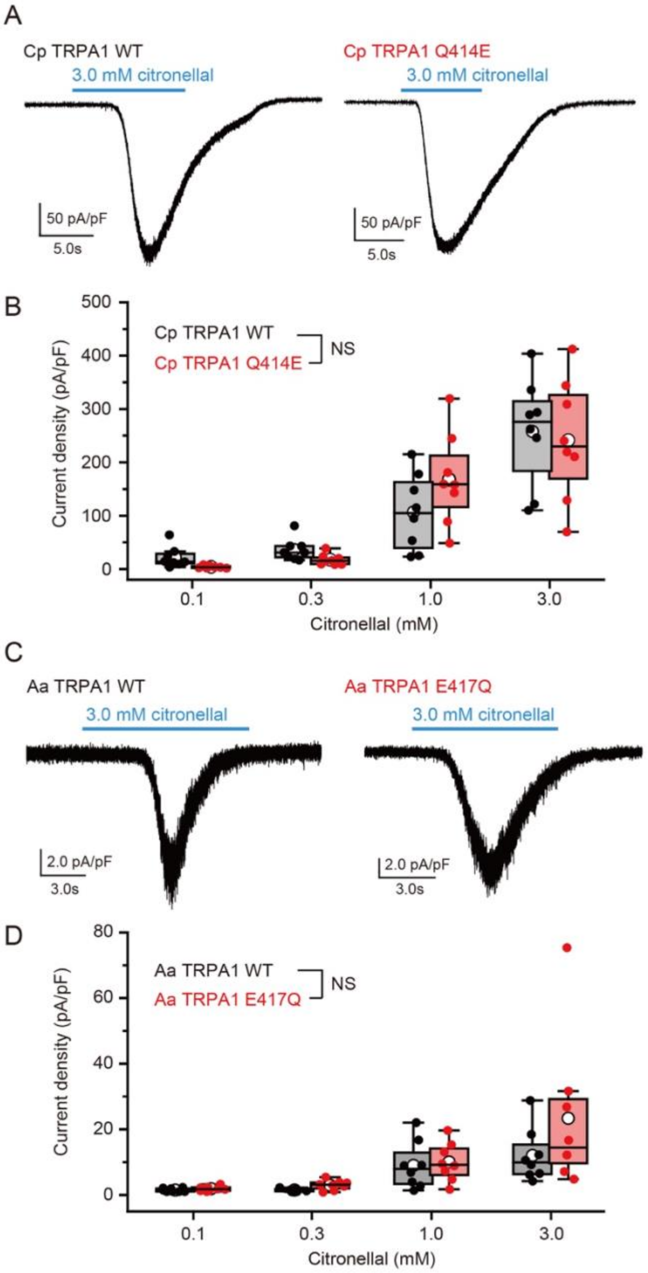

Supplement: Nguyen et al. SI Table 1 [file mmc6.pdf]
