# Supplementary material for: Single amino acids set apparent temperature thresholds for heat-evoked activation of mosquito transient receptor potential channel TRPA1
Source: J Biol Chem. 2022 Jul 16;298(9):102271. doi: 10.1016/j.jbc.2022.102271 (PMC9396403; doi:10.1016/j.jbc.2022.102271)

SI Figure 5. Representative current and temperature traces and Arrhenius plots for point mutants of mosquito TRPA1. Corresponds to Figure 2. Below each current trace, the corresponding temperature trace is shown. Each Arrhenius plot is derived from the corresponding current trace.

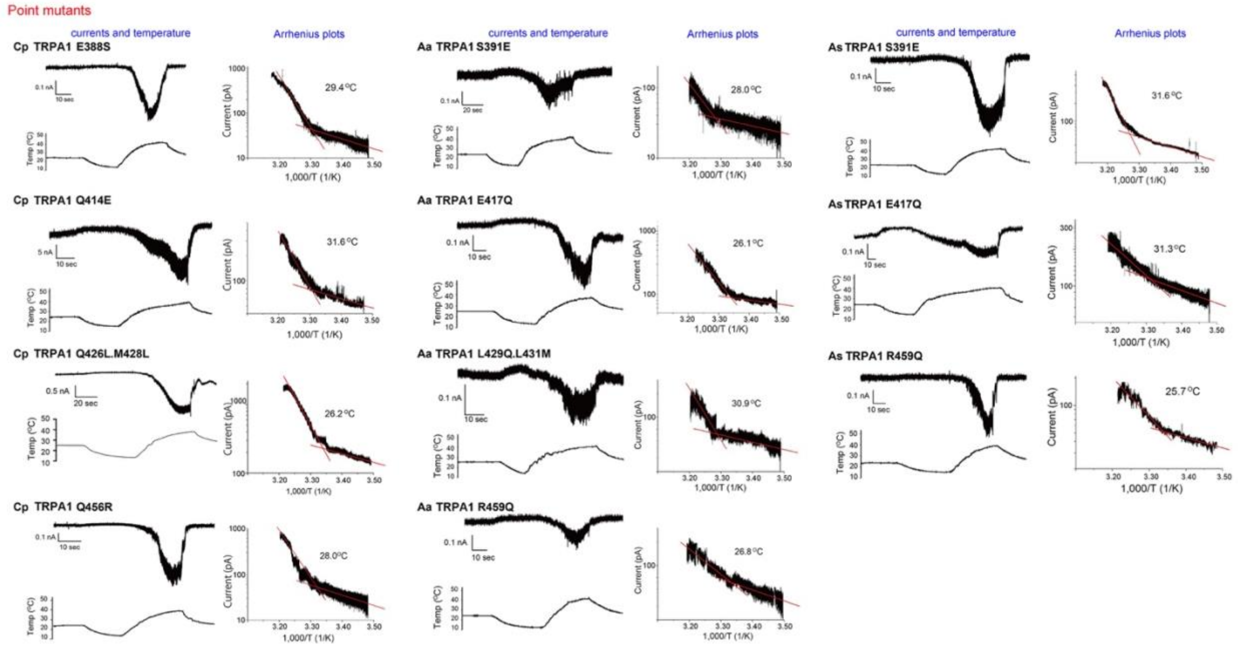

Supplement: Nguyen et al. revised SI Table 2 [file mmc7.pdf]
